# No Time for Myself: Personality Moderates Associations Between Solitude and Parental Well-Being

**DOI:** 10.1093/geroni/igaf122.494

**Published:** 2025-12-31

**Authors:** Theresa Pauly

**Affiliations:** Simon Fraser University, Vancouver, British Columbia, Canada

## Abstract

Parental well-being is often challenged by the demanding nature of caregiving responsibilities, leaving little opportunity for solitude (i.e., time to oneself). While prior research highlights the benefits of solitude for overall well-being, less is known about how these effects vary based on personality. Solitude may be especially important for parents who are introverted, to help recharge, or high in neuroticism, to manage heightened emotional reactivity. This study aimed to investigate whether personality traits moderate the association between solitude and daily well-being (as indicated by negative affect and cortisol levels) among parents. Data are from 318 parents of underage children (Mage=40.06 years; 45% male, 81% White) in the Refresher Daily Diary subproject of the Midlife Development in the United States Survey. Over 8 days, participants reported daily opportunities for time to themselves, negative affect, and provided saliva samples on days 2–5 to measure cortisol levels. Data were analyzed using multilevel modeling. On days on which participants reported having had time to themselves, they reported lower levels of negative affect (b = -0.05, p = .001) and steeper wake-evening cortisol slopes (b = -0.07, p = .011). Individuals with higher levels of neuroticism showed stronger associations between time to themselves and negative affect (b = -0.06, p = .007) as well as cortisol slopes (b = -0.09, p = .041). Facilitating moments of solitude for parents, especially those high in neuroticism, could serve as an effective strategy for supporting their emotional and physiological health.

